# Transcriptomic Analysis of Large Yellow Croaker (*Larimichthys crocea*) during Early Development under Hypoxia and Acidification Stress

**DOI:** 10.3390/vetsci9110632

**Published:** 2022-11-12

**Authors:** Yabing Wang, Run Chen, Qian Wang, Yanfeng Yue, Quanxin Gao, Cuihua Wang, Hanfeng Zheng, Shiming Peng

**Affiliations:** 1 Key Laboratory of Marine and Estuarine Fisheries, Ministry of Agriculture, East China Sea Fisheries Research Institute, Chinese Academy of Fishery Sciences, Shanghai 200090, China; 2 Marine Fisheries Development Center of Xiapu, Xiapu 355100, China; 3 College of Life Science, Huzhou University, Huzhou 313000, China

**Keywords:** *Larimichthys crocea*, hypoxia stress, acidification stress, transcriptome analysis

## Abstract

**Simple Summary:**

The large yellow croaker is one of the most economically important fish in China. In recent years, the deterioration of the water environment and unregulated aquaculture have caused great economic losses to the large yellow croaker breeding industry. The aim of this study was to analyze the effects of hypoxia and acidification stress on large yellow croaker. This study revealed that hypoxia and acidification stress suppressed the growth of the large yellow croaker. Transcriptome analysis revealed that genes of the collagen family play an important role in the response of large yellow croaker to hypoxia and acidification stress. The study elucidates the mechanism underlying the response of large yellow croaker to hypoxia–acidification stress during early development and provides a basic understanding of the potential combined effects of reduced pH and dissolved oxygen on Sciaenidae fishes.

**Abstract:**

Fishes live in aquatic environments and several aquatic environmental factors have undergone recent alterations. The molecular mechanisms underlying fish responses to hypoxia and acidification stress have become a serious concern in recent years. This study revealed that hypoxia and acidification stress suppressed the growth of body length and height of the large yellow croaker (*Larimichthys crocea*). Subsequent transcriptome analyses of *L. crocea* juveniles under hypoxia, acidification, and hypoxia–acidification stress led to the identification of 5897 differentially expressed genes (DEGs) in the five groups. Gene Ontology and Kyoto Encyclopedia of Genes and Genomes enrichment analyses revealed that several DEGs were enriched in the ‘protein digestion and absorption’ pathway. Enrichment analysis revealed that this pathway was closely related to hypoxia and acidification stress in the five groups, and we found that genes of the collagen family may play a key role in this pathway. The zf-C2H2 transcription factor may play an important role in the hypoxia and acidification stress response, and novel genes were additionally identified. The results provide new clues for further research on the molecular mechanisms underlying hypoxia–acidification tolerance in *L. crocea* and provides a basic understanding of the potential combined effects of reduced pH and dissolved oxygen on Sciaenidae fishes.

## 1. Introduction

The ocean is a complex ecosystem and alterations in oceanic environmental conditions affect the survival of marine organisms. At present, the ocean is under several threats and oceanic ecology has altered significantly in recent years. In addition, the marine environment is worsening on a gradual basis. The extensive anthropogenic use of fossil fuels has led to the release of CO_2_, which has been linked to a drop in ocean pH and the exacerbation of acidification, according to the Assessment Report by the IPCC [1]. The effect of ocean acidification on marine life has been studied extensively. The findings demonstrate that ocean acidification has a negative effect on the life processes of calcified organisms, including corals [2,3], mollusks [4], and crustaceans [5], and has a detrimental effect on the early embryonic stage and skeletal development of fishes [6,7,8].

It has been reported that ocean acidification damages the sensory organs, such as otoliths [9,10], of certain fishes, which exhibit signs of retardation [11]. Regions with coastal aquaculture are frequently anoxic owing to excessive animal breeding. Previous studies have demonstrated that aquatic animals exhibit slow movement, decreased feeding, and other characteristics when the amount of dissolved oxygen (DO) decreases in aquatic environments. These manifestations affect the normal activities of aquatic animals and can be fatal in several cases [12].

Respiration is inextricably linked to both acidification and hypoxia; therefore, the dynamics of acidification and hypoxia in ocean ecosystems are highly similar [7,13,14,15,16,17]. Acidification and hypoxia are observed in temperate coastal zones during the warmer months when the rates of respiration are highest and thermal stratification is most pronounced. The levels of acidification and hypoxia are extreme in regions that receive excessive nutrient loads, such as regions close to large coastal cities [16,18] or areas within eutrophic river plumes [14]. Therefore, studies investigating whether hypoxia and acidification stress affect the activities of aquatic animals have gained increasing attention in recent years.

The large yellow croaker (*Larimichthys crocea*) is an economically important marine animal, and the breeding technologies for *L. crocea* are highly advanced in China. The China Fishery Statistical Yearbook published in 2022 states that China produced 254,224 tons of large yellow croaker through mariculture in 2021 [19]. The Guanjingyang of Ningde is one of the primary sea areas in China for the spawning and migration of large yellow croaker and is also a significant breeding ground for this species of fish [20]. The high-density extensive breeding mode has a significant negative impact on sea ecology and poses a potential threat to the breeding of large yellow croaker. This is attributed to the growing number of farmers and the expansion of breeding in the Ningde Sea area in recent years [21]. The high number and density of aquaculture cages in this marine environment substantially hampers water flow exchange, which results in the gradual deposition of leftover bait, excrement, and other organic debris produced during aquaculture that are not removed by the tides. The pH of the water body consequently decreases and the levels of DO are reduced, which exposes the large yellow croaker to acidification and hypoxia stress [14,16,19]. Transcriptome analyses of hypoxic large yellow croaker brains have revealed a new aspect of neuro-endocrine–immune/metabolism regulatory networks that may help the fish avoid cerebral inflammatory injury and maintain energy balance after hypoxia stress [22]. *L. crocea* triggers a variety of processes within tissue (blood, gills, and liver) cells to adapt to hypoxia and reoxygenation environments, including adaptive regulation of energy metabolism, oxygen transport, and ion homeostasis, along with the involvement of various signaling pathways [23]. Transcriptome profiles of spleen and head kidney from hypoxic *Larimichthys crocea* have been performed, showing multiple immune-relevant pathways [24].

Transcriptome technologies have been integrated with high-throughput sequencing approaches as a routine and important strategy in recent studies for investigating the response of aquatic organisms to environmental stress [22,23,24,25,26,27]. In this study, we examined the differences in the transcriptomic expression among the five groups of large yellow croaker at 27 days of age. A series of differentially expressed genes (DEGs) were identified, and enrichment analysis revealed that the DEGs were significantly enriched in the ‘protein digestion and absorption’ pathway for coping with hypoxia and acidification stress in *L. crocea*. These findings provide important insights into the response of the large yellow croaker to hypoxia and acidification stress.

## 2. Materials and Methods

### 2.1. Fish Specimens

Fertilized eggs of large yellow croaker were obtained by artificial insemination from a commercial fish haven at Fuding, Ningde, Fujian Province, China.

### 2.2. Experimental Design

The DO at the suffocation point of large yellow croaker juveniles is reported to be 2.27 mg/L, and the appropriate DO content is 4.7 mg/L or higher [28]. The DO of the experimental group under hypoxia stress was reported to be 3.5 mg/L. The IPCC (2014) has predicted that ocean acidification may reduce the pH of seawater by 0.31 units in 2100. The pH of the experimental group exposed to acidification stress was therefore set to 7.3.

A total of four groups were considered in the breeding experiments, including the control group (normal group, N107; DO = 7.0 mg/L, pH = 8.1), hypoxia group (H107; DO = 3.5 mg/L, pH = 8.1), acidification group (A107; DO = 7.0 mg/L, pH = 7.3), and hypoxia–acidification group (double-factors group, D107; DO = 3.5 mg/L, pH = 7.3). The method used for controlling the DP and the pH is described hereafter.

The value of DO in seawater was reduced by flowing N_2_ gas through seawater, and the DO value was monitored in real time using an YSI Pro Solo water quality meter. The flow of N_2_ gas was continued until the DO dropped to the required level. The air flux of the hypoxia–acidification group was strictly controlled during the breeding experiment to ensure that the air flux was equal to the oxygen consumption in seawater. The pH of seawater was reduced by flowing CO_2_ into the seawater, and the pH was monitored in real time using an YSI 10 water quality meter. Similar to the flow of N_2_, the flow of CO_2_ was continued until the pH dropped to the required value.

With the exception of the DO and the pH, the other parameters remained the same. The temperature of the seawater was regulated at 23.0 °C and the specific gravity of seawater was maintained at 1.022. Each group comprised a total of 4 parallel controls, so 16 breeding buckets were used in this study. A total of 4.0 × 10^4^ fertilized eggs were placed in each breeding bucket.

### 2.3. Daily Management

The DO and pH values were measured every 15 min following the initiation of the experiments, and the parameters were adjusted over time if they underwent marked fluctuations. When the values of DO and pH reached stability, they were altered and measured every 6 h. The dead eggs and dead fish were removed following the hatching of the fertilized eggs to avoid polluting the seawater. The seawater was exchanged once every three days during the development of large yellow croaker into the larval stage, and one-third of the volume of seawater used for aquaculture was replaced each time. The values of DO and pH were pre-adjusted for the experimental groups during exchange, and the seawater was subsequently added to the breeding buckets.

The breeding buckets were supplemented with an oyster meal after the third day of hatching of the fertilized eggs of large yellow croaker. Briefly, 300 g of fresh oysters were blended in a food processor on a daily basis, and the blend was repeatedly washed and filtered with a 200-mesh sieve tulle. A small amount of the oyster blend was evenly poured into each of the breeding buckets several times. Feeding was initiated with artemia larvae after the sixth day of breeding, at a feeding density of approximately 45 larvae/mL. The larvae of large yellow croaker were fed 1–2 times on a daily basis based on the density of artemia larvae in the breeding bucket until the end of the breeding experiment. A total of 18 samples of juvenile large yellow croaker were selected from each group on the 27th day of hatching and divided into 3 equal parts. All the samples were immediately frozen in liquid nitrogen before storage at −80 °C until RNA extraction.

### 2.4. RNA Extraction, Quality Estimation, and Sequencing

The RNA was extracted using a sample RNA extraction kit by TIANGEN (Beijing, China). In order to ensure the quality of the samples used for transcriptome sequencing, the purity, concentration, and integrity of the RNA samples were determined using a Nanodrop 1000 spectrophotometer (NanoDrop Technologies, Wilmington, DE, USA), a Qubit 2.0 fluorometer (Invitrogen, Waltham, MA, USA), and an Agilent 4200 system (Agilent Technologies, Santa Clara, CA, USA). An Illumina mRNA-Seq Prep Kit was used for preparing the RNA samples for the RNA-seq libraries, which were subsequently sequenced by paired-end sequencing on an Illumina HiSeq 2000 sequencing platform (Illumina, San Diego, CA, USA).

### 2.5. Analysis of DEGs

In this study, the clean reads were aligned with the designated reference genome using the HISAT2 software (http://daehwankimlab.github.io/hisat2 accessed on 25 March 2021) for determining their respective positions on the reference genome. The expected number of fragments per kilobase of transcript sequence per million base pairs sequenced (FPKM) values for gene expression in each of the samples were determined using the featureCounts software 2.0.3 (https://sourceforge.net/projects/subread/ accessed on 25 March 2021).

The differences in gene expression between two groups were conducted by the strict Poisson distribution algorithm. The DEGs were defined as genes with absolute values of log_2_Ratio >1 and padj <0.05 (edgeR 3.6.1 (https://mirrors.tuna.tsinghua.edu.cn/CRAN/src/base/R-3/R-3.6.1.tar.gz accessed on 26 March 2021)). The ClusterProfiler software (version 3.4.4) was used for GO enrichment and KEGG pathway analyses of the DEGs. Based on the results of GO functional annotation, Fisher’s Exact Test was used for determining the significance of the differences between the control and treatment groups for identifying the enriched functional categories of the DEGs (*p* < 0.05). KEGG pathway enrichment analysis is similar to GO enrichment analysis; the KEGG pathways serve as the units while the reference genome serves as the background, and Fisher’s Exact Test is used for analyzing and calculating the level of significance of gene enrichment for each pathway, with the aim of identifying the significantly enriched metabolic and signal transduction pathways. TF annotation was additionally performed for the DEGs based on the annotation data from the Pfam database (http://pfam.xfam.org/ accessed on 26 March 2021) combined with the data for TF families in the DBD TF prediction database (http://bioinfo.life.hust.edu.cn/AnimalTFDB/#!/ accessed on 26 March 2021). The StringTie software (https://ccb.jhu.edu/software/stringtie/index.shtml accessed on 26 March 2021) was used to splice the mapped reads based on the sequence of the selected reference genome. The results were compared with the original genome annotation information in the GTF file using the GffCompare utility (http://ccb.jhu.edu/software/stringtie/gffcompare.shtml accessed on 26 March 2021) in GFF for determining the original unannotated transcription area and identifying new transcripts and novel genes.

## 3. Results

### 3.1. Effects of Hypoxia and Acidification Stress on the Length and Height of L. crocea

**Figure 1:**
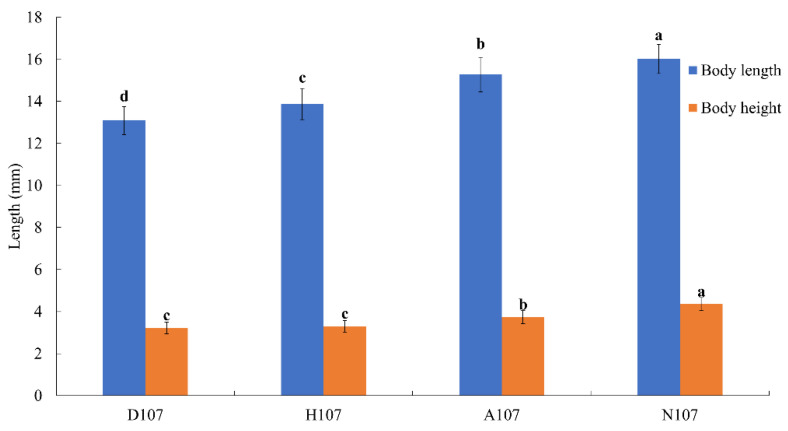
Body length and height of *L. crocea* in the different treatment groups on Day 27. Bars with different letters were considered significant at *p* < 0.05.

There were significant differences in the body length and height of *L. crocea* among the treatment groups after 27 days of hypoxia and acidification stress (*p* < 0.05) (Figure 1). Hypoxia and acidification stress in the A107 and H107 groups inhibited the increase in body length and height of *L. crocea*. The effect of hypoxia stress on the length and height of *L. crocea* was greater than that of acidification stress (*p* < 0.05). The inhibition of the increase in body length was aggravated by dual hypoxia–acidification stress in *L. crocea* (D107 group) (*p* < 0.05); however, the inhibition of the increase in body height was not significant (*p* < 0.05).

### 3.2. Transcriptome Profiles and Annotation

The RNA library of *L. crocea* was sequenced on an Illumina HiSeq 2000 sequencing platform. As depicted in Table 1, a total of 540,828,586 raw reads were generated, and approximately 38.9-50.6 M clean reads were obtained from each library. The short reads in the RNA-seq data were mapped to the genome of *L. crocea* using the HISAT2 program. The percentage of uniquely mapped transcripts ranged from 86.45% to 87.59%, while the percentage of transcripts with multiple mapping results ranged from 4.41% to 4.93%. A stringent set of *L. crocea* RNA transcripts comprising 29,417 annotated protein-coding genes was finally constructed.

### 3.3. Correlation Analysis between Samples

**Figure 2:**
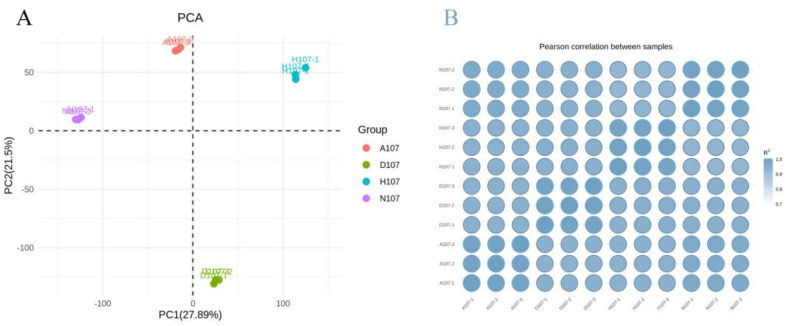
Analysis of the relationship between the samples under hypoxia and acidification stress in *L. crocea*. (**A**) PCA of the different transcriptome samples; (**B**) heatmap depicting the inter−−sample correlation coefficients.

The results of the principal component analysis (PCA) of the transcriptome samples indicated that the nature of the stress was the primary factor that affected transcriptomic expression. The sequenced samples were divided into four sub-categories based on the four types of treatment (Figure 2A). Heatmap analysis revealed that the results of clustering were similar to those obtained by PCA (Figure 2B).

### 3.4. Analysis of DEGs

Multiple comparisons of the transcriptome results and subsequent in−−depth analyses of the DEGs were performed for analyzing the dynamic alterations in *L. crocea* during hypoxia and acidification stress. The results of five groups (D107 vs. A107; D107 vs. H107; D107 vs. N107; A107 vs. N107; and H107 vs. N107) were compared and analyzed.

**Figure 3:**
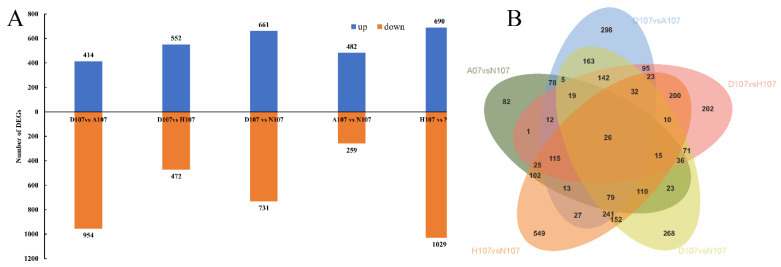
Number of DEGs under hypoxia and acidification stress in *L. crocea* depicted using (**A**) a histogram and (**B**) a Venn diagram.

The number of DEGs identified in each group are depicted in Figure 3A. The results demonstrated that acidification stress had the least effect while hypoxia stress had the greatest effect on *L. crocea*. The effect of dual hypoxia–acidification stress was lower than that of acidification stress on *L. crocea*. A total of 5897 DEGs were screened from the five groups (Figure 3B). A total of 26 DEGs were identified in the five groups of which only 17 DEGs were found to be associated with specific functions. The relative expression levels of these 17 DEGs are depicted in Table 2.

### 3.5. Function Enrichment of DEGs

**Figure 4:**
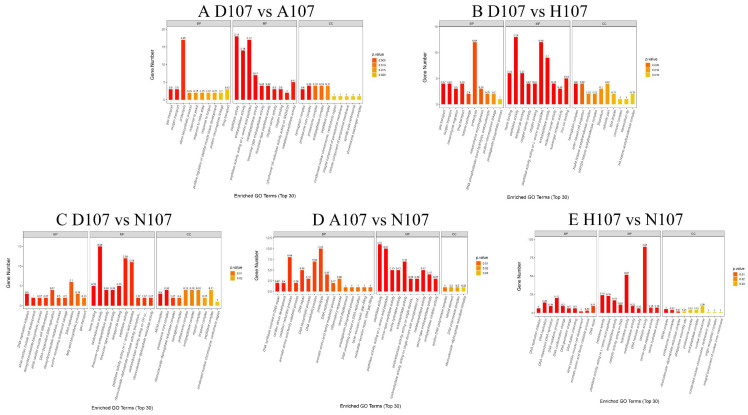
GO enrichment analysis of the DEGs under hypoxia and acidification stress.

The different functions of the DEGs were determined by Gene Ontology (GO) classification and Kyoto Encyclopedia of Genes and Genomes (KEGG) pathway analyses. In this study, the 10 most significantly enriched GO terms belonging to three major categories, namely the biological process, molecular function, and cellular component categories, were selected and displayed in a histogram. The genes that were differentially expressed between the D107 and A107 groups were enriched in the ‘proteolysis’ (17), ‘peptidase activity’ (18), and the ‘proteasome core complex’ (4) terms in the biological process, molecular function, and cellular component categories, respectively. The genes that were differentially expressed between the D107 and H107 groups were enriched in the ‘proteolysis’ (12), ‘peptidase activity’ (13), and the ‘hemoglobin complex’ (4) terms under the biological process, molecular function, and cellular component categories, respectively. The DEGs between D107 and N107 were enriched in the ‘DNA replication’ (6), ‘oxidoreductase activity’ (15), and the ‘proteasome core complex’ (4) terms under the biological process, molecular function, and cellular component categories, respectively. The DEGs between A107 and N107 were enriched in the ‘proteolysis’ (10), ‘peptidase activity’ (11), and ‘epsilon DNA polymerase complex’ (1) terms under the biological process, molecular function, and cellular component categories, respectively. The DEGs between H107 and N107 were enriched in the ‘DNA metabolic process’ (20), ‘catalytic activity’ (90), and ‘nuclear chromosome’ (9) terms under the biological process, molecular function, and cellular component categories, respectively (Figure 4). The genes that were differentially expressed between D107 and A107 or H107 were significantly enriched in the ‘proteolysis’ and ‘peptidase activity’ terms. The DEGs between N107 and D107, A107, or H107 were enriched in different GO terms.

**Figure 5:**
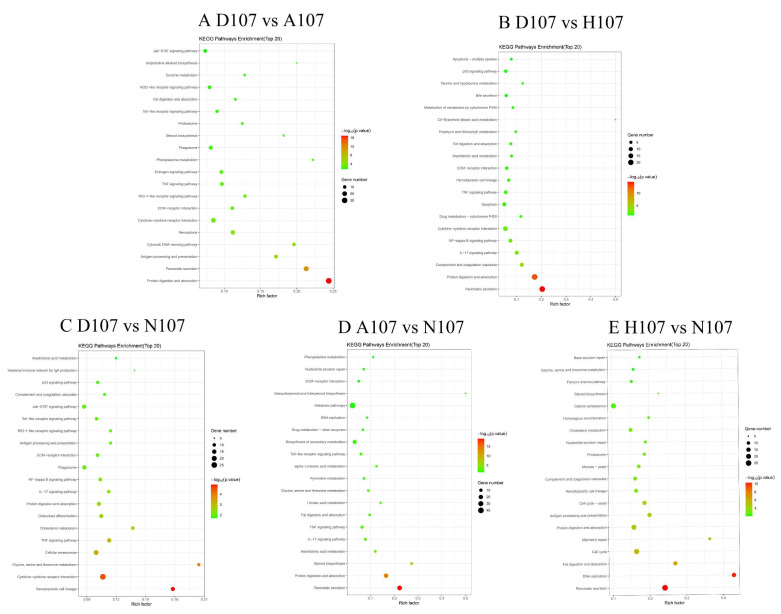
KEGG enrichment analysis of the DEGs under hypoxia and acidification stress.

**Figure 6:**
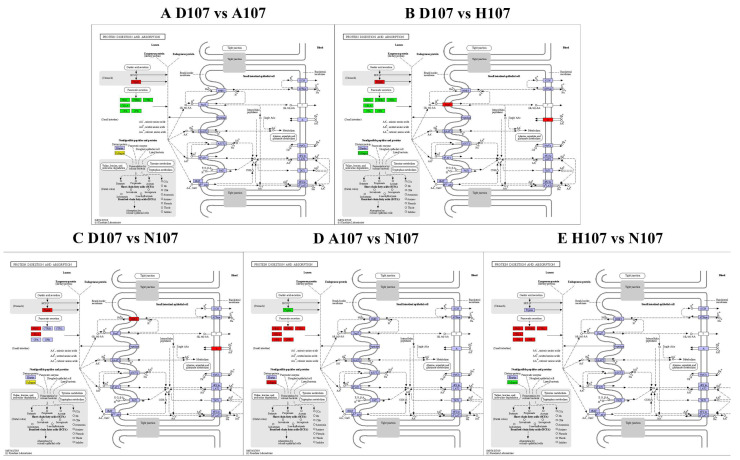
The ‘protein digestion and absorption’ pathway in KEGG was enriched under hypoxia and acidification stress.

The DEGs were mapped to the typical reference pathways in the KEGG database for analyzing and determining the significantly enriched signal transduction pathways and metabolic pathways of the DEGs. The genes differentially expressed between D107 and A107 and between D107 and H107 were enriched in 197 and 179 pathways, respectively. The DEGs between D107 and N107 and between A107 and N107 were enriched in 204 and 167 pathways, respectively. The genes that were differentially expressed between H107 and N107 were enriched in 227 pathways. The DEGs between D107 and A107 or H107 were enriched in the ‘protein digestion and absorption’, ‘pancreatic secretion’, ‘cytokine–cytokine receptor interaction’, ‘ECM-receptor interaction’, ‘TNF signaling pathway’, and ‘fat digestion and absorption’ pathways. The genes that were differentially expressed between N107 and D107, A107, or H107 were enriched in ‘glycine, serine, and threonine metabolism’ and ‘protein digestion and absorption’ pathways (Figure 5). These results indicated that the two pathways play a crucial role in the entire transcriptional context under hypoxia and acidification stress. At the same time, we observed that the DEGs of all the groups were significantly enriched in the ‘protein digestion and absorption’ pathway (Figure 6). The findings revealed that the ‘protein digestion and absorption’ pathway was the most important pathway for coping with hypoxia and acidification stress in *L. crocea*.

### 3.6. Analysis of Transcription Factors (TFs) and Prediction of Novel Genes

Comparison of the different treatment groups revealed that the DEGs were enriched in different TFs across the groups (Table S1). Comparison of the D107 and A107 groups revealed the DEGs were enriched in 23 TF genes belonging to 9 TF families, of which the DEGs were mainly enriched in the zf-C2H2 TF, followed by the HLH and homeobox TFs. The DEGs between D107 and H107 were enriched in 25 TF genes belonging to 33 TF families, and were mainly enriched in the zf-C2H2, homeobox, and HLH TFs. The DEGs between D107 and N107 were enriched in 27 TF genes belonging to 10 TF families, of which the HLH, zf-C2H2, and bZIP_2 TFs were mainly enriched. The DEGs between A107 and N107 were enriched in 9 TF genes belonging to 6 TF families, of which the HLH, bZIP2, and bZIP_Maf TFs were mostly enriched. The DEGs between A107 and N107 were enriched in 14 TF genes belonging to 6 TF families, of which the zf-C2H2, forkhead, and homeobox TFs were mainly enriched.

A total of 1,711 novel genes were identified by comparing the RNA-seq data with the reference genome annotation information. Functional annotation of the new transcripts with GO analysis revealed that the transcripts were primarily enriched in the ‘integral component of membrane’ (152 DEGs, cellular component), ‘ATP binding’ (104 DEGs, molecular function), ‘zinc ion binding’ (78 DEGs, molecular function), ‘nucleic acid binding’ (50 DEGs, molecular function), and ‘RNA-dependent DNA biosynthetic process’ (40 DEGs, biological process) terms (Table S2).

## 4. Discussion

Previous studies have investigated the sensitivity of the early developmental stages of three forage fish species, *Menidia menidia*, *M*. *beryllina*, and *Cyprinodon variegatus*, to hypoxia and acidification stress [6,7], and the results demonstrated that the sensitivity varies across the three species. The findings revealed that acidification stress reduces the post-hatching survival of *M*. *beryllina* but not that of the other two species, while hypoxia stress reduces the survival of both *M*. *menidia* and *M*. *beryllina* but not of *C*. *variegatus*. The effects of dual hypoxia–acidification stress were additive in *M*. *beryllina* but synergistically negative in *M*. *menidia*. However, the studies reported that *C*. *variegatus* is resistant to the combination of both stressors. The larval growth of *M*. *beryllina* is negatively affected by hypoxia or acidification stress, while dual hypoxia–acidification stress has an additive negative effect. The growth of the larval stages of *M*. *menidia* and *C*. *variegatus* is sensitive to hypoxia stress but not to acidification stress, and the combination of both the stressors has additive negative effects [6,7]. A study by Miller et al. also indicated that acidification stress increases fish mortality under hypoxia both directly and indirectly by increasing their vulnerability to predation via increased aquatic surface respiration [8]. In this study, the growth and development of large yellow croaker were suppressed under hypoxia and acidification stress at an early stage of development. The effects of hypoxia stress on the length and height of *L*. *crocea* were significantly higher than those of the fish under acidification stress, and the effect of dual hypoxia–acidification stress on body length was more significant than that of hypoxia stress alone. This finding further verified that the negative effect of dual hypoxia–acidification stress on fish growth is more pronounced compared to that of a single stress factor.

The number of DEGs under hypoxia stress was the highest, followed by that under dual hypoxia–acidification stress and acidification stress, compared to that of the control group. Although the number of DEGs in the group under dual hypoxia–acidification stress was not the highest, dual stress has the greatest effect on the growth of *L*. *crocea*. These findings suggested that DEGs could have a greater effect on the development of *L*. *crocea*.

The DEGs in all the five comparison groups were enriched in the ‘protein digestion and absorption’ KEGG pathway, which was subsequently studied in further detail. The pathway regulates the digestion and absorption of proteins [29,30,31] and also participates in other physiological functions. The ‘protein digestion and absorption’ pathway is involved in the response of *Epinephelus coioides* to *Pseudomonas plecoglossicida* infection [32], *Ctenopharyngodon idellus* following treatment with enrofloxacin [33], and the response of *Trachinotus ovatus* larvae to temperature stress [34]. In our previous studies we also demonstrated that hypoxia and acidification stress affect the non-specific immunity and antioxidant capacity of *L*. *crocea* [35].

The advancement of data mining techniques enabled the identification of genes closely related to the ‘protein digestion and absorption’ pathway. Notably, the collagen (collagen family), PRSS (trypsin), and CELA (pancreatic elastase II and pancreatic endopeptidase E) genes were identified in all the five groups. In this study, we emphasized the functionality of the collagen protein. Collagens are a family of related proteins that are characterized by the repeating Gly-X-Y tripeptide sequence, in which X frequently represents proline and Y frequently represents hydroxyproline, and the triple-helical structure [36]. The expression of collagen genes is regulated in the developmental stage in a tissue-specific manner and in response to a variety of biological and pharmacological inducers [37]. Previous studies have demonstrated that collagen is closely related to bone development [38,39]. Disruption of the transcription, translation, and synthesis of collagen genes can lead to a series of bone-related diseases [40,41,42]. In addition, collagens are also involved in extracellular matrix (ECM)−receptor interactions in focal adhesion complexes and the metabolic phosphatidylinositol 3′−kinase (PI3K)−Akt signaling pathway. The ECM is a three-dimensional scaffold comprising collagen, fibronectin, and several other proteins [43,44] and plays an important role in regulating cellular proliferation, differentiation, adhesion, migration, apoptosis, body development, and the establishment and maintenance of homeostasis. The abnormal regulation of the ECM is also closely related to osteogenesis imperfecta, chondrodysplasia, and other diseases [45,46,47]. Focal adhesion complexes comprise biological macromolecules and play an important role in the information transfer between cells and the ECM in cell survival, adhesion, motility and apoptosis, bacterial invasion of non-phagocytic cells, healing of wound tissues, and tumor metastasis [48,49]. The PI3K-Akt signaling pathway controls essential cellular processes, including transcription, translation, proliferation, growth, and survival and is activated by a variety of cellular stimuli or toxic insults [50,51,52]. In this study, both acidification and hypoxia stress affected the expression of collagens, indicating that acidification and hypoxia stress not only affected the development of bones but also the immune-related functions of *L*. *crocea*.

Transcriptome sequencing enables the detection of TFs and prediction of novel genes [53,54]. It has been demonstrated that TFs function as effector molecules in a wide variety of processes, including the regulation of gene expression, cellular functions, and environmental responses [54,55]. In this study, the DEGs were found to be enriched in a total of 125 TFs, including zf-C2H2, HLH, and homeobox, of which the zf-C2H2 TF was mostly abundant (Table S1). The zf-C2H2 protein regulates the expression of nearby genes by directly binding to the DNA. Previous studies have demonstrated that zf-C2H2 might be related to a variety of physiological processes and stress responses [56]. The results of this study are consistent with these reports and demonstrated that the zf-C2H2 TF may play an important role in the response of *L*. *crocea* to hypoxia and acidification stress.

The identification of novel genes can supplement and enrich the original genome annotation information and aid in mining new transcripts. These new transcripts could be lncRNAs or fusion transcripts. Fusion transcripts resulting from gene fusions, including chromosomal rearrangements, intergenic splicing, and lncRNAs, have been successfully used for the diagnosis, prognosis, and treatment of certain diseases [57]. In this study, a total of 1711 novel genes were identified in *L*. *crocea* under hypoxia and acidification stress. These novel genes allow a better understanding of the mechanism by which large yellow croaker respond to hypoxia and acidification stress.

## 5. Conclusions

In this study, the length and height of large yellow croaker were determined and comparative transcriptome analysis was performed for the first time to explore the differences in juvenile large yellow croaker under hypoxia and acidification stress. The findings revealed that hypoxia and acidification stress inhibited the growth of body length and height. Functional enrichment analysis of the DEGs revealed that the ‘protein digestion and absorption’ pathway was most significantly enriched in response to hypoxia and acidification stress. Mining of the differential genes in this pathway revealed that genes in the collagen family may play a key role in this pathway. The study also revealed that the zf-C2H2 TF may play an important role in response to hypoxia and acidification stress. In addition, a few novel genes have been identified and reported in this study. The study elucidates the mechanism underlying the response of large yellow croaker to dual hypoxia–acidification stress in the early developmental stage and provides fundamental information regarding the potential combined effects of reduced pH and DO on Sciaenidae fishes.

## Figures and Tables

**Table 1:** The raw, clean, total mapped, unique mapped, and multiple mapped reads obtained by RNA-seq analysis of *L. crocea* in different groups.

Group	Raw Reads	Clean Reads	Total Mapped Reads	Multiple Mapped Reads	Unique Mapped Reads
A107-1	51,350,116	50,634,408	46,499,266 (91.83%)	2,486,038 (4.91%)	44,013,228 (86.92%)
A107-2	39,767,726	39,188,754	35,953,671 (91.74%)	1,909,427 (4.87%)	34,044,244 (86.87%)
A107-3	42,103,238	41,554,092	38,194,333 (91.91%)	1,937,008 (4.66%)	36,257,325 (87.25%)
D107-1	47,421,264	46,738,380	42,831,314 (91.64%)	2,306,149 (4.93%)	40,525,165 (86.71%)
D107-2	44,541,626	43,825,858	39,986,358 (91.24%)	2,100,541 (4.79%)	37,885,817 (86.45%)
D107-3	44,308,218	43,637,372	39,926,492 (91.50%)	2,112,779 (4.84%)	37,813,713 (86.65%)
H107-1	39,377,942	38,901,524	35,751,378 (91.90%)	1,802,194 (4.63%)	33,949,184 (87.27%)
H107-2	41,232,648	40,666,410	37,285,801 (91.69%)	1,790,844 (4.40%)	35,494,957 (87.28%)
H107-3	45,168,584	44,621,350	41,053,873 (92.00%)	1,967,968 (4.41%)	39,085,905 (87.59%)
N107-1	49,941,386	49,268,714	45,159,242 (91.66%)	2,341,067 (4.75%)	42,818,175 (86.91%)
N107-2	46,245,704	45,746,386	42,054,378 (91.93%)	2,154,007 (4.71%)	39,900,371 (87.22%)
N107-3	49,370,134	48,956,334	45,188,019 (92.30%)	2,344,150 (4.79%)	42,843,869 (87.51%)

**Table 2:** Comparative analysis of the 17 DEGs and their expression in the different groups.

Gene ID	Gene Name	log_2_FoldChange				
		D107 vs. A107	D107 vs. H107	D107 vs. N107	A107 vs. N107	H107 vs. N107
gene-papln	Proteoglycan-like sulfated glycoprotein	−1.00	−1.23	1.03	2.02	2.26
gene-LOC104921168	Microfibril-associated glycoprotein 4-like	3.14	2.25	1.25	−1.90	−1.01
gene-lamb3	Laminin subunit beta 3	1.11	1.22	2.71	1.58	1.49
gene-LOC113744908	Nebulin-like	−3.38	−2.98	−1.77	1.59	1.20
gene-LOC104937404	Lamc2 laminin, gamma 2	1.96	2.01	4.17	2.19	2.15
gene-LOC104930325	Collagenase 3	1.64	1.73	3.21	1.55	1.47
gene-LOC109139265	Complement C1q-like protein 2	2.28	6.45	4.33	2.04	−2.12
gene-LOC104927899	Cytosolic sulfotransferase 3	−1.17	3.04	−2.42	−1.27	−5.47
gene-LOC109140889	Endonuclease domain-containing 1 protein	2.02	1.93	3.89	1.86	1.95
gene-LOC104935020	Butyrophilin-like protein 2	−1.54	−1.77	−2.82	−1.29	−1.05
gene-LOC104929223	Proproteinase E-like	−1.14	−2.59	1.91	3.03	4.49
gene-LOC113744424	Collagenase 3-like	1.23	1.74	3.60	2.35	1.86
gene-LOC109141109	Rho-related GTP-binding protein RhoG-like	1.37	1.62	3.05	1.66	1.42
gene-LOC104922034	Elastase-1	−3.24	−5.16	−1.77	1.45	3.37
gene-hgfac	HGF activator	1.26	1.45	2.69	1.41	1.23
gene-LOC104937999	Growth-regulated alpha protein	3.96	1.83	5.19	1.21	3.35
gene-LOC104940478	Gastricsin	3.49	4.10	1.61	−1.89	−2.50

## Data Availability

The RNA-seq data have been submitted to the NCBI Short Read Archive (SRA) under accession number: PRJNA851972 (https://www.ncbi.nlm.nih.gov/sra/PRJNA851972 accessed on 12 July 2022).

## Supplementary Materials

The following supporting information can be downloaded at: https://www.mdpi.com/article/10.3390/vetsci9110632/s1, Table S1: Different transcript factors of DEGs were dissimilar in different treatment comparison; Table S2: GO terms for novel genes.
